# Utility of Polygenic Risk Scoring to Predict Cognitive Impairment as Measured by Preclinical Alzheimer Cognitive Composite Score

**DOI:** 10.14283/jarlife.2022.1

**Published:** 2022-02-23

**Authors:** Q. Gao, P. Daunt, A.M. Gibson, R.J. Pither, for the Alzheimer’s Disease Neuroimaging Initiative

**Affiliations:** 1 Cytox Limited, Manchester, UK.

**Keywords:** Polygenic risk, cognitive decline, Alzheimer’s disease

## Abstract

**Background:**

The utility of Polygenic Risk Scores (PRS) is gaining increasing attention for generating an individual genetic risk profile to predict subsequent likelihood of future onset of Alzheimer’s disease (AD), especially those carry two copies of the APOE E3 allele, currently considered at neutral risk in all populations studied.

**Objectives:**

To access the performance of PRS in predicting individuals whilst pre-symptomatic or with mild cognitive impairment who are at greatest risk of progression of cognitive impairment due to Alzheimer’s Disease from the Alzheimer’s Disease Neuroimaging Initiative (ADNI) as measured by the Preclinical Alzheimer Cognitive Composite (PACC) score profile. Design: A longitudinal analysis of data from the ADNI study conducted across over 50 sites in the US and Canada.

**Setting:**

Multi-centre genetics study.

**Participants:**

594 subjects either APOE E3 homozygotes or APOE E3/E4 heterozygotes who upon entry to the study were diagnosed as cognitively normal or with mild cognitive impairment.

**Measurements:**

Use of genotyping and/or whole genome sequencing data to calculate polygenic risk scores and assess its ability to predict subsequent cognitive decline as measured by PACC over 5 years. Results: Assessing both cognitively normal and mild cognitive impaired subjects using a PRS threshold of greater than 0.6, the high genetic risk participant group declined more than the low risk group over 5 years as measured by PACC score (PACC score reduced by time).

**Conclusions:**

Our findings have shown that polygenic risk score provides a promising tool to identify those with higher risk to decline over 5 years regardless of their APOE alleles according to modified PACC profile, especially its ability to identify APOE3/E3 cognitively normal individuals who are at most risk for early cognitive decline. This genotype accounts for approximately 60% of the general population and 35% of the AD population but currently would not be considered at higher risk without access to expensive or invasive biomarker testing.

## Introduction

Dementia describes an intra-individual pattern of decline in memory and thinking impairing at least two domains of cognition (1). Alzheimer disease (AD) is the most common cause of dementia. The majority of cases occur after age 65, constituting late-onset AD (LOAD), while cases occurring earlier than age 65 are considerably rarer, constituting less than 5% of all cases and are termed early-onset AD (EOAD) (2, 3). Approximately 1%–2% of AD is inherited in an autosomal dominant fashion (ADAD) and can present with very early age of onset and a more rapid rate of progression and is sometimes associated with other neurologic symptoms seen less frequently in sporadic AD (4). Sporadic or LOAD show a multifactorial heredity pattern caused by genetic and complex environmental interactions associated with several predisposing factors and age. The rate of cognitive deterioration during the development of AD varies among individuals (5, 6) and seems to be guided by a combination of genetic and environmental factors (7). Some genes, such as CLU, PICALM, and CR1, have been shown to be related to AD as indicated by genome-wide association studies (GWAS) (8, 9). However, only apolipoprotein E (APOE) polymorphisms have been established as consistent genetic susceptibility factors for LOAD in all populations studied in the world (10).

Development of polygenic risk scoring (PRS) algorithms that can capture all the genetic contribution towards the risk of developing AD (11) is an attractive strategy to allow for stratifying patients at risk prior to or as part of screening for clinical trial participation Furthermore understanding risk for future onset or progression of symptoms due to AD at a much earlier stage may lead to greater uptake of lifestyle interventions that have been shown to at least delay the progression of disease by several years. It is generally recognised that changes to lifestyle that will reduce risk for onset of AD are most effective when made earlier in life prior to any significant symptoms being displayed. A PRS test that can provide a cost-effective and widely accessible way of supporting the stratification of cognitively normal and MCI patients into those that are highest risk of developing AD will provide an additional tool for identifying individuals most likely to benefit from new disease modifying therapies or other patient management decisions.

Here we investigate the performance of our PRS in predicting cognitive decline with a particular focus on whether it can provide predictive information on identifying early changes of cognitive performance in cognitively normal individuals. As such, polygenic risk has been used here to predict cognitive changes using the modified PACC score (12, 13) over 5-year period. We have focussed on subjects who were either APOE E3 homozygotes and APOE E3/E4 heterozygotes (see Table 1-3). This accounts for approximately 80% of the general population but also that of the study population (sub-analyses of other APOE genotypes is compromised by low subject numbers).

**Table 1:** Characteristics of participants

**1a Characteristics of Participants**			
**Characteristics**	**MCI at**	**CN at Baseline**	**Total Group**
	**Baseline**		
Number	424	228	652
Age mean (SD)	72 (7.3)	75 (5.3)	73 (6.8)
Male/Female	248/176	115/113	363/289
PACC at baseline mean (SD)	-5.5 (3.9)	-0.05 (2.7)	-3.6 (4.4)
**1b Characteristics of CN and MCI Participants– APOE status**			
E2E4	12	2	
E3E3	216	158	
E3E4	158	62	
E4E4	38	6	
**1c Characteristics of CN and MCI Participants – E3E3 and E3E4**			
Number	374	220	
Age mean (SD)	73 (7.3)	75 (5.2)	
Male/Female	216/158	110/110	
PACC at baseline mean (SD)	-5.3 (3.7)	-0.05 (2.7)	
PRS negative (<0.6)	106	117	

**Table 2:** Participants carrying APOE E3E3 and E3E4 in CN Group

**2a Number of participants by APOE status**														
**APOE**	**bl**	**m0**	**m1**	**m2**	**m3**	**m4**	**m6**							
**Status**		**6**	**2**	**4**	**6**	**8**	**0**							
E3E3	158	157	152	145	83	124	71							
E3E4	62	60	60	59	29	47	26							
**2b Number of participants by APOE status and risk score**														
Characteristics	bl	bl	**m0**	**m0**	**m1**	**m1**	**m2**	**m2**	**m3**	**m3**	**m4**	**m4**	**m6**	**m6**
			**6**	**6**	**2**	**2**	**4**	**4**	**6**	**6**	**8**	**8**	**0**	**0**
APOE	E3E	E3E	E3E	E3E	E3E	E3E	E3E	E3E	E3E	E3E	E3E	E3E	E3E	E3E
Status	3	4	3	4	3	4	3	4	3	4	3	4	3	4
High	45	58	45	56	42	56	39	55	19	26	33	44	20	23
Low	113	4	112	4	110	4	106	4	64	3	91	3	51	3

bl: baseline; m: month. Thus month 6 is represented by m06

**Table 3:** Participants carrying APOE E3E3 and E3E4 in MCI group

**3a Number of participants by APOE status**														
**APOE**	bl	**m0**	**m1**	**m2**	**m3**	**m4**	**m6**							
**Status**		**6**	**2**	**4**	**6**	**8**	**0**							
E3E3	216	207	206	188	174	149	111							
E3E4	158	154	155	136	117	112	72							
**3b Number of participants by APOE status and risk score**														
**Characteristics**	**bl**	**bl**	**m0**	**m0**	**m1**	**m1**	**m2**	**m2**	**m3**	**m3**	**m4**	**m4**	**m6**	**m6**
			**6**	**6**	**2**	**2**	**4**	**4**	**6**	**6**	**8**	**8**	**0**	**0**
APOE	E3E	E3E	E3E	E3E	E3E	E3E	E3E	E3E	E3E	E3E	E3E	E3E	E3E	E3E
Status	3	4	3	4	3	4	3	4	3	4	3	4	3	4
High	110	158	105	154	104	155	99	136	90	117	82	112	59	72
Low	106	0	102	0	102	0	89	0	84	0	67	0	52	0

bl: baseline; m: month. Thus month 6 is represented by m06

## Methods

Data used in the preparation of this article were obtained from the Alzheimer’s Disease Neuroimaging Initiative (ADNI) database (adni.loni.usc.edu). The ADNI was launched in 2003 as a public-private partnership, led by Principal Investigator Michael W. Weiner, MD. The primary goal of ADNI has been to test whether serial magnetic resonance imaging (MRI), positron emission tomography (PET), other biological markers, and clinical and neuropsychological assessment can be combined to measure the progression of mild cognitive impairment (MCI) and early Alzheimer’s disease (AD).

ADNI genotyping and/or whole genome sequencing data was used to calculate polygenic risk scores and assess their ability to predict subsequent cognitive decline as measured by the modified PACC score over 5 years.

### Sample Description

In order to understand the predictive performance of the PRS algorithm above and beyond that which is provided for by APOE status alone, we initially investigate data from 652 CN and MCI subjects selected from ADNI 1, ADNI GO, ADNI 2 and ADNI 3 studies examined between 2005 and 2017 (see Table 1a). Due to the low sample sizes (n≤50) of APOE E2E4 and E4E4 individuals in either CN or MCI groups (see Table 1b) at baseline (bl), further analyses were only carried out in APOE E3 homozygotes and APOE E3/E4 heterozygotes. Therefore, all results shown in this paper were based on 594 CN and MCI subjects who were either carried two copies APOE E3 allele or were APOE E3/E4 heterozygotes (see Table 1c) and had modified PACC score data at entry to the study in addition to having suitable genetic data and at least 5 years’ worth of follow up cognitive testing and imaging scans.

### Genotyping Procedures and Quality Control

The ADNI samples were genotyped using with Whole Genome Sequencing and/or the Illumina Omni 2.5M BeadChip array. Quality control checks were performed using PLINK software (www.cog-genomics.org/plink/2.0/). Checks included the exclusion of SNPs with missingness greater than 0.02 and minor allele frequency of less than 0.01. SNPs with Hardy-Weinberg equilibrium p-value less than 1 x 10-6 were also excluded. After such checks 8,990,292 SNPs were left for analysis of which approximately 114,000 were used as part of the polygenic risk scoring algorithm (14).

### The ADNI modified PACC score

PACC is a composite score which combines tests that assess episodic memory, timed executive function and global cognition which has been shown to be able to detect the first signs of cognitive decline before clinical signs of MCI manifest (15). In this study, we use a ADNI modified PACC with Digit Symbol Substitution (mPACCdigit) (12, 13) downloaded using R package “adnimerge” (https://adni.bitbucket.io/reference/pacc.html#references).-.

In ADNI, Free and Cued Selective Reminding Test (FCSRT) is not used and has been replaced Delayed Recall test that is included within the Alzheimer’s Disease Assessment Scale (ADAS) as a suitable proxy to be included in the modified PACC score. Furthermore, mPACCdigit score also includes the Digit Symbol Substitution Test (DSST) when available (ADNI1) and mPACCtrailsB uses (log transformed) Trails B as a proxy for DSST. Raw component scores standardized according to the mean and standard deviation of baseline scores of ADNI subjects with normal cognition to create Z scores for each component (Z=(raw - mean(raw.bl))/sd(raw.bl)). The Z scores are reoriented if necessary, so that greater scores reflect better performance. The composite is the sum of these Z scores. At least two components must be present to produce a score. If more than two components are missing, the PACC will be NA.

### Calculation of Polygenic Risk Scores

A specifically built, proprietary software called SNPfitRTM was used for all subsequent PRS calculations. The PRS calculations are based on a pre-determined logistic regression model based on the modelling of the association between the incidences of variants within a large panel of SNPs with a known links to AD to the presence of the disease in a substantial cohort of subjects (Escott-Price et al.16). Subject age, sex and APOE status are included as covariates. The software calculates the normalised sum of the individual scores weighted by their effect sizes for each SNP, adds the values for the covariates and derives the predicted risk from the model equation.

Effect sizes were determined from the International Genomics of Alzheimer’s (IGAP) study. The score contribution from SNPs with missing values were imputed based on the population frequency of the effect allele for that SNP.

### Statistical Analysis

The polygenic risk scores generated were exported for the analysis presented.

R version 4.0.4 (https://www.r-project.org/) was used to carry out all data processing and analysis. The receiver operating characteristic (ROC) analysis and AUC calculations were performed using R package “pROC”. Modified PACC data were obtained from R package “adnimerge” (https://adni.bitbucket.io/reference/pacc.html#references). T tests were performed in R using the t.test() function to determine whether there is significant different between high and low risk groups (see p-value in Results).

To determine whether applying a PRS approach would provide further accuracy for predicting cognitive decline as measured by a modified PACC, we analysed the cognitively normal APOE E3/E3 and APOE E3/ E4 individuals, where both genetics and modified PACC score data were available (n=220, see Table 1c). PRS were calculated and individuals were assigned to either “high risk” (defined as a PRS ≥ 0.6, n=103) or “low risk” (PRS<0.6, n=117) groups (see Table 1c). A similar evaluation was performed on APOE E3/E3 and APOE E3/E4 individuals who entered the study with a diagnosis of MCI and for whom both genetic data and PACC score data were available (n=374 , See Table 1c). PRS were calculated and MCI individuals were assigned to “high risk” (defined as a PRS ≥ 0.6, n=268) or “low risk” (PRS<0.6, n=106) groups (see Table 1c). Note that not all subjects had follow-ups at each time point over the 5 years. Thus, the number of subjects varies at each follow-up check points. A PRS of 0.6 was chosen as a threshold based on an optimal balance between sensitivity and specificity in previous studies (17).

## Results

The overall performance for predicting individuals who would decline by at least -1 PACC score within 5 years from a baseline diagnosis of either cognitively normal or mild cognitive impairment was 65.6% (CI:61.3-69.8) area under the curve (AUC), suggesting PRS could be an effective stratification tool to identify patients with a higher likelihood to decline cognitively over a period of 5 years.

### PRS to predict early cognitive decline from a cognitively normal baseline

**Figure 1:**
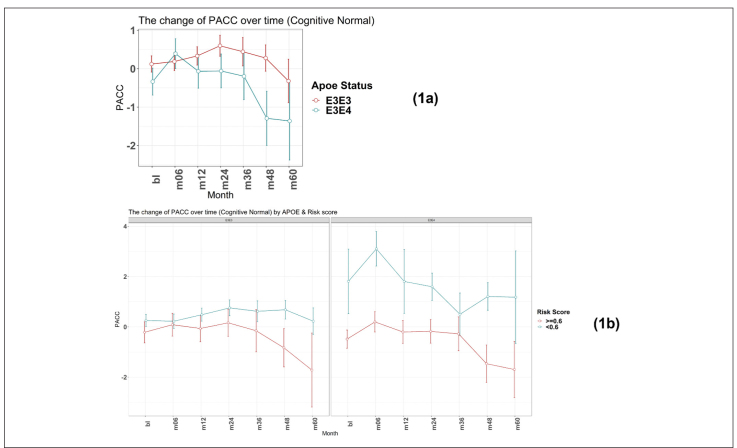
Time-course PACC scores for individuals carrying APOE E3E3 and E3E4 in CN Group: (1a) The change of PACC over time in individuals who entered as cognitively normal over 5-year period grouped by APOE status; (1b) The change of PACC over time in individuals who entered as cognitively normal over 5-year period grouped by risk score (bl=baseline, m=month)

As expected, as measured by modified PACC score, those individuals who carry a copy of the APOE E4 allele are more likely to decline cognitively than those who are APOE E3 homozygotes over a 5-year period (see Figure 1a). The mean change in modified PACC score in APOE E3/E3 after 60 months was just -0.4 points ±4.1 whereas APOEE3/E4 individuals declined, on average, by 1.3 points ±4.7, on the modified PACC score scale after 60 months (see Figure 1a).

There was a significant difference in the average change of the modified PACC score approximate to 2 between the two groups observed from as early as 48 months (high risk n=77, low risk n=94; high risk average PACC=-1.2, low risk average PACC=0.7; p-value =0.003, see Table 2). When considering APOE E3 homozygotes alone, the difference in the change of PACC score between the high risk and low risk groups observed was 2 points over 60 months years (high risk n=20, low risk n=51; high risk average PACC=-1.7, low risk average PACC=0.2, p=0.12, see Table 2). Importantly, though sample size is smaller (see Table 2), low PRS risk E3/E4 individuals that entered the study as cognitively normal appeared more likely to remain cognitively stable compared with the high risk group (Figure 1b).

### PRS to predict early cognitive decline from an MCI baseline

**Figure 2:**
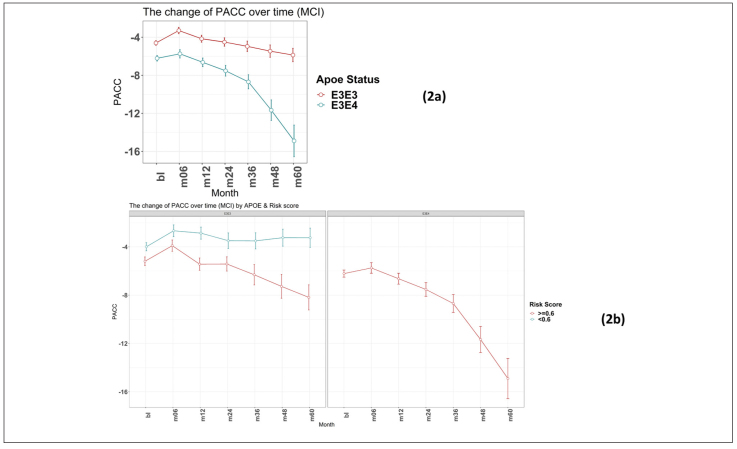
Time-course PACC scores for individuals carrying APOE E3E3 and E3E4 in MCI Group: (2a) The change of PACC over time in individuals who entered as MCI (EMCI or LMCI) over 5-year period grouped by APOE status; (2b) The change of PACC over time in individuals who entered as MCI (EMCI or LMCI) over 5-year period grouped by risk score (bl=baseline, m=month)

Again, as expected, those individuals carrying an E4 allele demonstrate greater cognitive decline, on average, compared to E3 homozygotes at all timepoints over the 5-year follow-up period (E3/E3 mean PACC change after 60 months -1.4 points ±5.6; E3/E4 mean PACC change after 60 months -8.9 points ±12.2; Figure 2a).

There were no individuals within the APOE E3/E4 MCI cohort (n=158) with a low PRS score (PRS <0.6). This is unsurprising, since these individuals who have already declined cognitively to an MCI diagnosis are likely to have a high PRS. Notwithstanding, this meant that a comparison between low and high PRS risk within the MCI group individuals could not be made. However, the APOE E3 homozygote MCI group contained both high PRS risk (≥0.6, n=110) and low PRS risk (<0.6, n=106) individuals (Figure 2b). Among this group, high PRS risk patients declined, on average, by approximately 1 point more than the low risk group after over 6 months (high risk n=105, low risk n=102; high risk average PACC=-3.9, low risk average PACC=-2.7, p=0.07, see Table 3) and a significant additional 5 points over 60 months (high risk n=59, low risk n=52; high risk average PACC=-8.2, low risk average PACC=-3.2, p-value<0.001, see Table 3) above those calculated as low risk, who did not decline further over the 5 year period studied (Figure 2b).

## Discussion

PRS approaches have demonstrated accuracies of between 75 and 84% for predicting onset of AD when including APOE status, sex and age in addition to PRS (16). In particular, the PRS approach as developed by Escott-Price et al., (14) is built as a sum of the weighted contributed of 10,000s of Single Nucleotide Polymorphisms (SNPs) where the weights are the β-coefficients of each SNP association with the disease. In contrast to other PRS algorithms, where fewer SNPs have been used (for example just 31 SNPs (18)) this approach includes SNPs that are not considered as having genome wide significance in GWAS studies. However, inclusion of this vastly increased number of variants which alone carry sub-threshold significance provides an additive contribution to the overall performance that may be substantive and also reduce risk that performance is not lost when being applied across different cohorts. Until now the analyses performed using this approach have been carried out to predict those individuals diagnosed with AD or MCI (19) versus those who are cognitively normal, though PRS algorithms have been used to look at a variety of AD pathology and risk by Altmann et al. (20). Patients who present to clinicians with very mild or subjective cognitive complaints can provide a diagnostic and patient management challenge in terms of decisions on whether to progress to more expensive and/or invasive testing or to discharge. Easier access to risk evaluation data will help better patient management decisions in a cost-efficient manner and provide further basis for dialogue on risk mitigation through lifestyle changes. Furthermore, screening of large pre-symptomatic populations to identify potential clinical trial participants for prevention studies in AD is challenging. Genetic risk prediction can be generated from DNA simply extracted from saliva or blood samples, thus providing a viable route to wide-scale risk stratification to characterise potential clinical trial subjects.

We have previously reported (17) on the performance of a PRS algorithm for predicting those individuals, with a bassline diagnosis of MCI who would decline by at least 15 ADAS-Cog13 points in 4 years with an AUC of 72.8% (CI:67.9-77.7) increasing to 79.1% (CI: 75.6-82.6) when also including those at baseline who were considered cognitively normal. Furthermore, by designating MCI patients as either high or low risk as determined by a PRS threshold of 0.6 it was observed that the high risk group declined, on average, by 1.4 points more on the CDR-SB scale than the low risk group over a period of 4 years. This performance in predicting cognitive decline due to AD was similar to that when defining risk using a pTau/ Ab1-42 ratio as measured in a cerebrospinal fluid (CSF) sample.

This study was designed to demonstrate the potential utility of a specific PRS algorithm for identifying individuals at highest risk of developing early or continued cognitive decline from either pre-symptomatic (CN) baseline or a relatively early stage of their disease (MCI). The results show the potential to use a PRS approach to identify those individuals most likely to decline cognitively. Importantly this includes identifying cognitively normal APOE E3 homozygous individuals who are at most risk for early cognitive decline due to AD. This genotype accounts for approximately 60% of the general population and 35% of the AD population but currently would not be considered at higher risk without access to expensive or invasive biomarker testing. PRS could therefore provide a useful tool for identifying individuals within this group who require additional monitoring, investigation or, with future developments, therapeutic intervention.

This study shows that PRS predictions can identify individuals with the highest risk of subtle cognitive decline, as measured by PACC scores, in patients who did not display any measurable symptoms upon entry to the ADNI study. The timeframe of 5 years used for the analysis is relevant in the context of both primary and secondary prevention trials and clinical practice. Furthermore, future work will be conducted to evaluate the predictive performance of our PRS algorithm in order to identify patients during mid-life (40-60 years old) at risk of future cognitive deficits due to AD which can provide a critical strategy for reducing the number. This genetic risk assessment represents an easily accessible intervention with the potential to reduce cost and patient burden through blood or mouth swab testing. Additionally, this genetic risk assessment provides an extremely valuable tool for expanding recruitment into secondary prevention trials which currently are typically limited to recruiting E4 carriers only. Furthermore, as disease modifying drugs enter clinical practice finding an easy to deploy risk prediction test to identify patients most likely to benefit from therapeutic intervention will be critical.

PRS does have its own challenges and limitations. For example, this work considers genetic risk together with age and sex in developing a model for predicting further development of cognitive symptoms but does not consider other risk factors that are known to influence onset and development of disease, for example, lifestyle and environment. Further studies will be required to combine both genetic and lifestyle risk factors to accurately identify those individuals at the most risk of Alzheimer’s disease.

## Study Limitations

This study is not without limitations, with sample size being the primary shortcoming. This was particularly relevant in evaluating the APOE E4 carrier sub-group (E2/E4, E3/E4 and E4 homozygous, see Table 1b). Furthermore, studies with larger sample sizes across all diagnostic categories, including those declining from a cognitively normal baseline, will be important to understand broader utility. As with most studies of this nature, observing similar performance in alternative cohorts is important and is critical towards the understanding and confirmation of polygenic risk score assessment for use in clinical trial recruitment and in clinical practice.
